# Supplementary material for: Responses in Plant Growth and Root Exudates of Pistia stratiotes under Zn and Cu Stress
Source: Plants (Basel). 2024 Mar 5;13(5):736. doi: 10.3390/plants13050736 (PMC10934204; doi:10.3390/plants13050736)
Supplement: Supplementary file 1 [file plants-13-00736-s001.zip › plants-2823816-supplementary.pdf]

Supplementary Materials

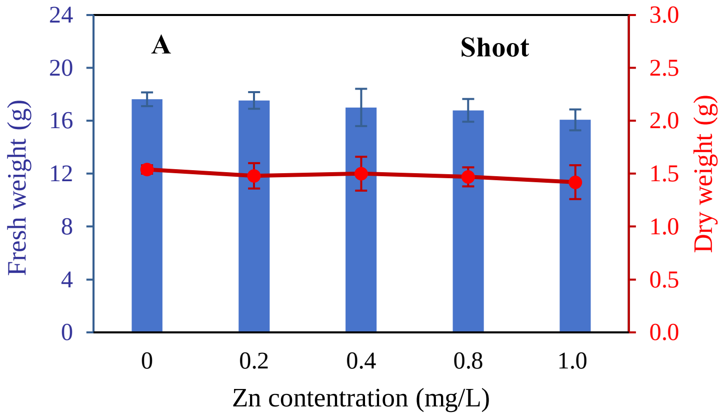

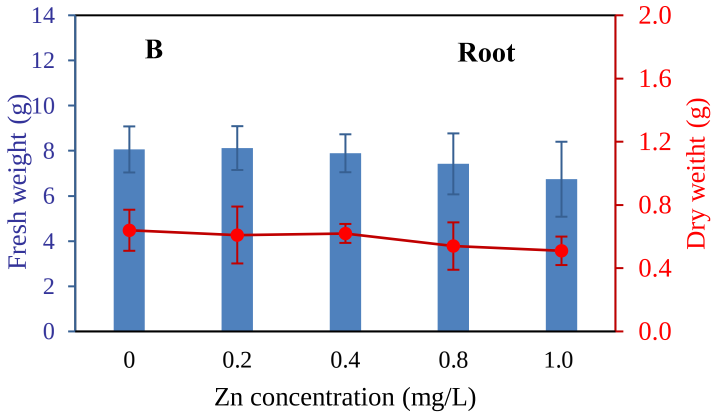

Figure S1. (**A**,**B**) The growth of *P. stratiotes* after exposure to the low concentrations of Zn (0, 0.2, 0.4, 0.8, 1.0 mg/L) for 20 days.

Table S1. Root exudate compounds and relative amount of *P. stratiotes*.

Compounds	Formula	Relative Amount (%)		
		CK	Zn	Cu
2−Phenylethanethiol, TMS derivative	C11H18SSi	0.2	—	—
3−Propylnorleucine	C9H19NO2	0.6	—	—
2,4−Di−tert−butylphenol	C14H22O	1.75	1.8	1.35
1−Heptatriacotanol	C37H76O	0.25	—	0.8
1−Pentanol, 2−ethyl−4−methyl−	C8H18O	3.45	4.3	4.25
E,E,Z−1,3,12−Nonadecatriene−5,14−diol	C19H34O2	0.2	—	0.2
1,4−Methanoazulen−3−ol, decahydro−1,5,5,8a−tetramethyl−, [1S−(1à,3á,3aá,4à,8aá)]−	C15H26O	—	0.25	—

1−Decanol, 2−hexyl−	C16H34O	—	0.25	0.6
1−Eicosanol	C20H42O	—	0.25	—
1−Hexadecanol	C16H34O	—	0.8	0.4
2−Hexyl−1−octanol	C14H30O	—	0.4	—
Azulene	C10H8	—	0.6	0.6
p−Xylene	C8H10	0.5	0.35	0.35
Benzene, 1,3−dimethyl−	C8H10	—	0.25	—
Phenol, 2,2'−methylenebis[6−(1,1−dimethylethyl)−4−methyl−	C23H32O2	20.45	14.35	14.2
17−Pentatriacontene	C35H70	1.7	1.2	0.8
7,9−Di−tert−butyl−1−oxaspiro(4,5)deca−6,9−diene−2,8−dione	C17H24O3	1.15	4.5	0.5
2,4−Dimethyl−5,6−dimethoxy−8−aminoquinoline	C13H16N2O2	—	0.25	—
tert−Hexadecanethiol	C16H34S	0.4	0.65	0.3
10−Octadecenal	C18H34O	0.25	—	—
Benzaldehyde, 2,4−dimethyl−	C9H10O	0.45	—	—
Hexanal, 2−ethyl−	C8H16O	0.85	—	—
5−Octadecenal	C18H34O	—	0.45	—
Benzaldehyde, 3,4−dimethyl−	C9H10O	—	0.55	0.45
Octadecanal, 2−bromo−	C18H35BrO	—	0.2	—
Eicosanal−	C20H40O	—	—	0.25
3−Hydroxypropyl palmitate, TMS derivative	C22H46O3Si	0.2	0.35	0.3
Fumaric acid, hexadecyl propargyl ester	C23H38O4	—	—	0.2
Carbonic acid, tetradecyl 2,2,2−trichloroethyl ester	C17H31Cl3O3	—	—	0.35
Bicyclo[4.4.1]undeca−1,3,5,7,9−pentaene	C11H10	0.5	0.45	—
Cyclopentane, 1−methyl−2−(4−methylpentyl)−, trans−	C12H24	—	0.2	0.2
Ethylbenzene	C8H10	—	—	0.25
Heptasiloxane, hexadecamethyl−	C16H48O6Si7	—	—	0.25
Hexadecane, 1−iodo−	C16H33I	—	—	0.4
Heptacosane, 1−chloro−	C27H55Cl	—	—	0.35
1−Cyclopentyleicosane	C25H50	0.2	—	—
1−Iodo−2−methylundecane	C12H25I	0.5	—	—
2−Methylhexacosane	C27H56	0.7	—	—
5,5−Diethylheptadecane	C21H44	0.2	—	—
Dodecane, 2,7,10−trimethyl−	C15H32	0.3	—	—
Eicosane, 2,4−dimethyl−	C22H46	0.2	—	—
Heneicosane, 11−(1−ethylpropyl)−	C26H54	0.35	—	—
Hexadecane, 1,1−bis(dodecyloxy)−	C40H82O2	0.8	0.5	0.65
Hexadecane, 2,6,11,15−tetramethyl−	C20H42	9.9	7.05	3.45
Nonadecane, 2−methyl−	C20H42	0.2	—	—
Octadecane, 3−ethyl−5−(2−ethylbutyl)−	C26H54	1.1	1.4	0.6
Pentadecane, 2,6,10,14−tetramethyl−	C19H40	0.25	—	—

Tetradecane, 2,6,10−trimethyl−	C17H36	0.4	0.6	0.8
Tetrapentacontane, 1,54−dibromo−	C54H108Br2	0.35	—	0.45
2,6,10−Trimethyltridecane	C16H34	—	1	0.35
2−Methylpentacosane	C26H54	—	0.2	0.2
5−Butyl−5−ethylheptadecane	C23H48	—	0.2	0.2
Docosane, 11−decyl−	C32H66	—	0.2	—
Dodecane, 2,6,11−trimethyl−	C15H32	—	0.8	1.25
Eicosane, 2−methyl−	C21H44	—	0.2	0.2
Heptadecane, 2,3−dimethyl−	C19H40	—	0.45	—
Hexadecane, 2,6,10,14−tetramethyl−	C20H42	—	0.3	0.3
Pentadecane, 2,6,10−trimethyl−	C18H38	—	0.2	0.2
Tetracosane, 11−decyl−	C34H70	—	0.2	—
Tetradecane, 2−methyl−	C15H32	—	0.25	—
Heptadecane, 9−octyl−	C25H52	—	—	0.25
N,N−Dimethyldodecanamide	C14H29NO	—	0.4	—
Arachidamide, N−ethyl−	C22H45NO	—	—	0.6
Deoxyspergualin	C17H37N7O3	—	—	0.5
Naphthalene, 2−methyl−	C11H10	—	—	1.35
Oleic acid, 3−(octadecyloxy)propyl ester	C39H76O3	0.8	—	—
Phthalic acid, di(2−propylpentyl) ester	C24H38O4	0.9	1.05	0.95
Digitoxin	C41H64O13	—	0.25	—
9−Octadecenamide, (Z)−	C18H35NO	—	1.25	1.05
Cyclopentane, undecyl−	C16H32	0.4	0.45	0.45
Dodecane	C12H26	2.1	2.05	1.5
Eicosane	C20H42	2.6	2	1.2
Heneicosane	C21H44	1.65	0.6	1.2
Heptadecane	C17H36	2.6	1.9	1.5
Hexadecane	C16H34	0.8	1.35	0.9
Pentacosane	C25H52	4.3	2.6	2.85
Pentadecane	C15H32	4.15	0.8	0.55
Tetracosane	C24H50	0.3	—	—
Tetradecane	C14H30	2.25	2.7	3.15
Nonadecane	C19H40	—	—	0.7
Acetic acid n−octadecyl ester	C20H40O2	0.7	0.85	0.65
Carbonic acid, eicosyl vinyl ester	C23H44O3	0.2	—	—
Dichloroacetic acid, tetradecyl ester	C16H30Cl2O2	0.45	—	—
Phthalic acid, hept−4−yl isobutyl ester	C19H28O4	0.5	0.6	0.6
Toluene−4−sulfonic acid, 2,7−dioxatricyclo[4.3.1.0(3,8)]dec−10−yl ester	C15H18O5S	—	0.2	—
1,3−Benzenedicarboxylic acid, bis(2−ethylhexyl) ester	C24H38O4	—	—	0.2
Didecan−2−yl phthalate	C28H46O4	—	—	0.2
Eicosyl heptafluorobutyrate	C24H41F7O2	—	—	0.25

Octatriacontyl pentafluoropropionate	C41H77F5O2	—	—	0.25
Dibutyl phthalate	C16H22O4	0.85	0.75	0.85
1b,4a−Epoxy−2H−cyclopenta[3,4]cyclopropa[8,9]cycloundec[1,2−b]oxiren−5(1aH)−one, 2,7,9,10−tetrakis(acetyloxy)decahydro−3,6,8,8,10a−pentamethyl−	C28H38O11	0.3	—	—
Octasiloxane, 1,1,3,3,5,5,7,7,9,9,11,11,13,13,15,15−hexadecamethyl−	C16H50O7Si8	—	0.4	—
Triacontyl acetate	C32H64O2	—	0.2	—
à−L−Fucopyranose 1,2:3,4−bis(benzeneboronate)	C18H18B2O5	—	0.3	—
9−Octadecenenitrile, (Z)−	C18H33N	—	—	0.2
2,6,10−trimethylundecanoic Acid, 2,2,2− trifluoroethyl ester	C16H29F3O2	—	—	0.2
